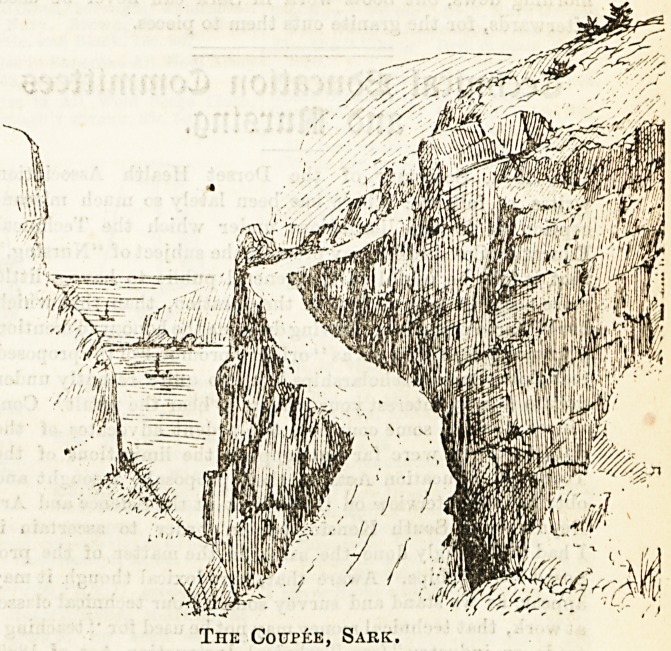# The Hospital Nursing Supplement

**Published:** 1894-05-12

**Authors:** 


"The Hospital" Nursing Mirror.

Being the Extra Nursing Supplement of "The Hospital" Newspaper.

[Contributions for this Supplement should be addressed to the Editor, The Hospital, 428, Strand, London, W.C., and should have the word "Nursing" plainly written in left-hand top corner of the envelope.]

### News from the Nursing World.

### "THE HOSPITAL" CONVALESCENT FUND.

Subscriptions to The Hospital Convalescent Fund for Nurses should be sent to the Hon. Secretary, at 428, Strand, as soon as possible. In compliance with the wishes of some of our readers the managers of the Fund have decided to allow suitable applicants, as far as possible, to choose their own place of rest. This arrangement must commend itself to many tired or invalided nurses for whom a long railway journey is undesirable. The first consideration will be given to urgent cases, and nurses should fully explain their inability to get needful change of air without the aid of the Fund in making application to the Hon. Secretary.

### AN EXCELLENT ASSOCIATION.

The Ladies' Sanitary Association has published its thirty-sixth annual report, and the Secretary mentions that a number of subscriptions still remain unpaid. An increase in donations has kept the society out of debt, although the balance in hand is but small. The sanitary tracts issued by the Association have been widely circulated during the year.

### MRS. BREWER'S AFTERNOON.

A most comprehensive entertainment is being organised for Thursday afternoon, June 7th, at the Queen's Hall, in aid of four good objects—the North-Eastern Hospital for Children, the Cripple Branch of the Ragged School Union, the Blind in their Homes under Mrs. Starey, and the Poor of Spitalfields. A vast amount of sympathy ought to be aroused by such diverse and deserving charities, and no doubt Mrs. Brewer, who is organising the entertainment, will be as successful as heretofore in her appeal to the generosity of the public. It is proposed to reserve the whole of the orchestra for nurses in uniform, who will be admitted at a shilling each to the performance, which will include recitations by well known authors, an excellent concert, &c. The name of the Baroness Burdett-Coutts heads the list of patrons.

### THE NURSES' HOTEL.

The Royal Avenue, Chelsea, only a few minutes' walk from Sloane Square, is a very pleasant and quiet street to live in, and Miss Culverhouse's Nurses' Hotel or Home, at No. 18, is certainly well located. The house is convenient, not only for private nurses working on their own account, but for country visitors who desire inexpensive accommodation, either with or without board, in cubicles or single rooms.

### THE LIVING AND THE DEAD.

At a recent meeting of the Leicester Board of Guardians an increase in the payments of the present staff of nurses was discussed. Miss Ellis is to be congratulated on having called attention to two points, viz., the doubtful wisdom of giving high salaries to untrained women and the even more important question of the night nursing. According to the *Leicester Daily Post* Miss Ellis has certainly good grounds for considering one nurse insufficient for the infirmary for she relates that five deaths occurred during one night last winter. It is obvious that to do her duty reverently and decently to five dying or dead paupers would sorely tax the powers of even a well-trained nurse. In the meantime all the other patients would have to be practically unheeded. "Urgent" cases rightly demand immediate attention, but "serious" ones need it almost more, because a little timely help and skilled nursing may not only save them much suffering but possibly lead eventually to restoration to health. The claims of the dying are paramount, but surely the Leicester Guardians, in acknowledging this incontrovertible fact, will also make due provision for proper night nursing of the sick and the helpless who are not yet quite close to the gates of death.

### HOW NOT TO ACT IN EMERGENCIES.

An exhaustive inquiry was very properly made by the coroner with regard to the recent death of a child at Coventry. A poor little girl of four, already suffering from measles, accidentally set her nightgown on fire, and was so badly burnt that death eventually resulted. A doctor was sent for when the catastrophe occurred, and by his advice the child was taken to the Coventry and Warwickshire Hospital. It was there refused admittance on account of the measles, and sent on to the Fever Hospital, where the matron could not take it in without an order from the medical officer. The burnt child was thereupon taken back home, and its wounds were at last dressed by the doctor who had been attending it for measles. According to the local press just indignation was evinced at the inquest at the inhumanity of the little girl's wounds being allowed to remain undressed for two or three hours whilst she was bandied about from one place to another. Oil and old rags are not difficult of attainment in any locality, and a first dressing of some sort should have been immediately used. As an ordinary fly was requisitioned for the conveyance of this infectious case, surely it might have occurred to some one that a hospital nurse might also enter, and there and then apply such dressing and coverings of cotton wool as would not only have been merciful to the unfortunate child, but would have removed from all concerned the slur of incompetency in dealing with ordinary emergency cases.

### INEFFICIENT OR EDUCATED?

The registration of midwives is a subject which has been already discussed at considerable length on a great many occasions, but a new theory appears to have been evolved recently by the gentleman who remarked at a committee meeting on April 10th, reported by the *British Medical Journal*, that "the totally inefficient women, in his experience, were very useful, and very rarely did very much mischief; in fact, hardly did any at all." However, for the consolation of qualified midwives and well trained nurses, it is perhaps desirable to add that "a good deal of dissent was expressed from the opinion that untrained midwives did not cause a great deal of danger and loss." At a meeting of the South Wales and Monmouthshire branch of the British Medical Association in April the following encouraging resolution was passed: "That this meeting recognises the necessity, in the interests of the public, for legislation to secure the proper education, registration, and supervision of midwives, and upholds the principles advocated by the Midwives' Registration Association that the education and registration of midwives should be under medical control and supervision."

### OF AN UNCERTAIN AGE.

"Mrs. Henry stated that she did not know her age, not possessing a certificate, and her parents being dead," reports the local press regarding the person selected by the Guardians to hold the position of nurse in Sligo Workhouse. The other two applicants owned to thirty-eight and thirty-nine years respectively. The matter of training was not, apparently, even mentioned by the Board at the meeting when the appointment was made, but whilst the applicants who owned to thirty-nine summers received three votes, the woman who "did not know" was the fortunate recipient of nineteen.

### A NURSE FOR BUILTH.

A public meeting was recently held at Builth to consider the question of the institution of a trained district nurse for the sick poor of the town. The scheme was unanimously agreed to, and a committee has been formed to carry it out.

### THE PROPOSED CONVALESCENT HOME.

The proposed Dr. Samuel Johnstone Moore Convalescent Home for Nurses already referred to in our columns, to be erected in the neighbourhood of Glasgow, is intended, says the *Scotsman*, "to be used as a temporary home for nurses whose health has temporarily broken down in the exercise of their profession, and who (having always borne an excellent character, as to which the certificate of the Matron for the time being of the Glasgow Training Home for Nurses . . . . shall be sufficient evidence) having been for a period of at least twelve years subsequent to the completion of their probationary training directly connected with and under the control of the Glasgow Training Home for Nurses or any institution of a similar character with which it may hereafter be incorporated or amalgamated." If there are not enough temporarily invalided nurses who fulfil these conditions, others who have done four years' work in connection with the Glasgow Training School may be admitted to the proposed home for short periods, and, if space still remains, nurses from other Glasgow hospitals may be admitted if they have accomplished the stipulated length of service and are of irreproachable character.

### WHO IS THE BEST JUDGE?

The Local Government Board are trying to secure a properly trained nurse for the sick poor in the Ballinrobe Union and Infirmary, and they have refused to sanction the appointment of Miss M'Loughlin on the ground of her not being qualified. The *Western People* reports that the Local Government Board has written "requesting the Guardians to proceed to a new election, and to offer at the same time such a salary as would induce properly qualified candidates (trained) to come forward." But in the face of this excellent advice the master reported that Miss M'Loughlin had been allowed to take up the duty, and then a resolution was proposed and carried which, for originality, it would be difficult to rival, the chief argument (?) advanced being that the former occupant of the office was untrained, and is now the matron. The appeal of the Local Government Board to have the paupers nursed by a properly paid and properly trained woman has found no favour, and the attitude at present adopted by the Guardians towards the Local Government Board may be gathered from a speech which is thus reported: "We will give them trouble enough. They may dissolve the Board and send down paid guardians before we give in."

### MELBOURNE.

The Melbourne District Nursing Society has held its ninth annual meeting, Sir John Madden presiding on the occasion. The urgent need for subscriptions was pointed out, the balance remaining to the credit of the society being extremely small. The past year showed a report of increased work without a proportionate increase of income. Three nurses are constantly employed, one of them specially devoting herself to the midwifery cases. The work of all of these appears to be so highly valued that there seems little fear of the association eventually failing to receive the support so urgently demanded.

### A FANCY PICTURE.

With the somewhat comprehensive title of "The Nursing System," our valued contemporary *The Queen* had an article in last week's issue which gives a somewhat limited and inaccurate impression of the position held by trained nurses to-day. The writer evidently considers that intelligent trained women are still the conventional "ministering angels" which it was the fashion to call the gentle smoother of the sufferer's pillow in a more sentimental age than the present. But the writer of the article in *The Queen* is probably unaware that no nurse worthy of her name would agree with the description of the time spent in the exercise of her chosen life's work as "her dreary hours." Those are possibly the sentiments held by an amateur or a three months' probationer, but not by the qualified nurse. The question of earnings is also attacked in the same article with very imperfect knowledge of the subject. No certificated nurse whose record is good need accept the starvation wages quoted, and certainly any well-trained woman has merely herself to blame if she takes an engagement in any private institution where nurses receive only "a third, or in many instances even a quarter" of the fees charged for her services. There is a paragraph in the article on "District Nursing" which appears this week in another column of The Hospital which shows very clearly the improved financial position held by trained nurses of to-day.

### SHORT ITEMS.

Two nurses have charge of 156 patients between them at West Ham Workhouse.—Dr. Cook has offered to give a weekly lecture to the nurses at Hampstead Workhouse Infirmary.—A district nurse is to be established in Coleraine in affiliation with the Queen's Jubilee Institute.—The Kendal Home Nursing Association has held its first annual meeting.—Silver medals have recently been awarded to Nurse Emily and Nurse Jeanette for five years' faithful work on the staff of the Blackheath and Richmond Nursing Institution.

### On General Nursing.

By Rowland Humphreys, M.R.C.S., L.R.C.P.Lond.

XI.—PNEUMONIA (*continued*).

Emphysema, due to rupture of the lung, occurs sometimes, where there has been old disease of the tissue, as by tubercule. The substance of the lung gets inflated. Empyæma, as a consequence of local suppuration, also occurs; in some cases the abscess bursts into a bronchus, in others it burrows its way through the front wall of the chest, appearing at or near the lower end of the sternum. In most cases it has to be opened and drained. The chances of ultimate recovery are very remote. Delirium tremens occurs in this as in other acute diseases, and augurs very badly for the patient. The condition of the heart has to be especially watched. Acute rheumatism and acute nephritis occasionally occur.

There is a great tendency to fresh attacks in those who have once had this complaint, especially among young and old people. In the latter there may be no symptoms of the complaint, except those made out on physical examination, as the fever is often absent.

Sometimes meningitis comes on during the complaint; at other times the patient has an attack of acute mania.

Acute pneumonia is also called croupous and lobar pneumonia, from the fibrinous exudation, and from the large area affected, respectively. It occurs twice as often in men as in women, and attacks adults up to middle age most commonly. In most cases no cause can be assigned. Several bacteria have been found in close connection with the complaint, but there is no definite proof that they are or are not the cause of it. The disease runs in epidemics, and it varies greatly as to the death-rate in different years, different places, and different epidemics. Following it comes a danger of phthisis and of chronic or fibroid pneumonia.

*Treatment.*—The signs of the disease in the stage of hyperæmia are still unmarked, or at least the diagnosis can only be one of probability. The fever being high the patient must be kept in bed, and as much at rest as the delirium and restlessness of fever will permit, in order to save the heart as much as possible in view of the strain which the fully-developed complaint will throw upon it. The vomiting, which is so often present, will prevent any large amount of food being given at one time, but small quantities, repeated every two hours or so, will be of great service, especially if it does not contain too much albuminous nourishment. Milk and soda water, or milk and barley water, cream, iced drinks, and iced or hot beef-tea made in the ordinary way may be given. As a drink barley water and lemon, with the whites of two eggs added to each pint, will be found acceptable. It is also nourishing. Peptonised foods are also excellent. The great thing is to give as much nourishment as can be digested and absorbed by the patient within a reasonable time after taking it, and not to give it oftener than every two hours. More annoyance is given and more danger is incurred at the present time by overfeeding than by anything else. If the stomach be overfilled it will press on and hinder the movements of the heart; while if the food is given too frequently the stomach gets no rest, and either rejects the food or fermentation takes place, and, the products being absorbed, palpitation and other complications ensue. The author has before now seen a patient nearly killed by a nervous wife. The patient had a distended stomach, and that peculiar gurgling noise which accompanies fermentation and overdistension was very audible. A few hours' complete intermission from food pulled him round wonderfully, and the Cheyne-Stokes breathing (a peculiar, rhythmical irregularity of respiration), from which he was suffering, passed away at the same time.

The pain in the side may be relieved by strapping, by bandaging, by poulticing, and at the early part of this stage small doses of opium in the form of Dover's powder are often administered.

On the first suspicion of pneumonia the patient should be placed in a steam tent, which should reach to his knees, at least if an adult; if a child it should surround the entire bed. The atmosphere in the tent should be kept at a temperature of between 60 and 65 deg. Fah. Above this temperature the heat gets rather trying; below it the cough gets more troublesome, the expectoration less, the cyanosis more marked (meaning that the blood is not being aerated so well as it should), and the patient is apt to become very drowsy from the condition of the blood. In the steam-kettle some antiseptic should be placed, as pneumonia under some circumstances is highly infectious, and then it appears to be due to a bacillus, whose development may be checked, perhaps, by the constant inhalation of some poisonous (to it) drug. The delirium and sleeplessness should be combated, and if the latter be attacked early, and the patient saved the exhaustion which results from want of sleep, far fewer cases of heart failure will be seen. At any rate some strong measures will have to be taken when the exhaustion begins to tell on the respiratory centres, as shown by the breathing becoming quicker, and the pulse slowing at the same time. And in cases when the pulse has fallen during this restless stage to below 110 the matter becomes serious, and an injection of morphia is then the best and quickest remedy. Opium in all its forms has the unfortunate power of suppressing the expectoration if given in sufficient quantity, and there is a danger that through this power, which prevents the proper aeration of the blood, as also through its direct power of causing sleep, the blood will get into such a condition that the patient may sink into a hopeless state of coma.

The expectoration is to be assisted by drugs, such as ipecacuanha, and if much blueness makes its appearance carbonate of ammonia and other stimulants are often useful. Alcohol is to be used only in case of great exhaustion, and in cases where there is an element of delirium tremens. If the tongue becomes moister under its influence, and the pulse slower and stronger, it is doing good; in the opposite case it is doing harm.

The fever has to be met by the administration of the various drugs which have the property of reducing it; and of these quinine, antifebrin, antipyrin hold the first place, though, of the two latter each has its own dangers; the former causing the face, &c., to become very blue; the latter depressing the heart.

Another method of reducing the fever is by cold (at 60 deg. Fah.), or tepid (at 80 deg. Fah.), sponging, or bathing, or wet packing, or the use of Leiter's tubes, through which passes a current of water at the temperature of the air, or ice may be added, and the temperature of the coil thus further reduced. One of these methods should be employed each time the fever rises over 103 deg. Fah. A stimulant should be given both before and after bath or sponging. The patient will often take food immediately after the bath, when he had previously refused it, so that the nurse should always be prepared for this.

### Novelties for Nurses.

Mr. Robert French, 118, Trongate, Glasgow, wholesale watch factor and jeweller, has sent us his catalogue containing full particulars of a great variety of watches of many designs and prices, rings, brooches, bangles, &c. Mr. French has a manufactory in Switzerland, and copies of the catalogue can be had on application.

### The Advantages and Privileges of a Trained District Nurse and the Duties of the District Towards Her.

By Henry C. Burdett.

Eighteen years ago, when Miss Florence Lees first established the Metropolitan and National Nursing Association, her ideas were subjected to severe criticism, mainly on two grounds. First, it was urged that the idea of attempting to confine the members of the nursing staff to gentlewomen was ridiculous, because they were not likely to offer themselves in sufficient numbers, and if they did they would be physically unfit to discharge many of the duties ; indeed, Miss Sherriff and Miss Frances Power Cobbe went so far as to say, when nursing was advocated as a career for gentlewomen, that men who held such views ought to be withstood, and that they belonged to the class which usually beat their wives.

Secondly, it was said that to call the Association Metropolitan and National was high-sounding nonsense, and that it was highly improbable that such a title would ever be justified in practice. Mr. Rathbone, M.P., who has probably done more for nursing in this country than any other man, supplied evidence at the recent annual meeting that Miss Florence Lees' Association is to-day both metropolitan and national, and that the Queen's Jubilee Institute had charged it with the responsible duty of training the Queen's nurses. Hence Mrs. Dacre Craven is to be congratulated upon the prescience and courage with which she stood to her guns eighteen years ago, and upon her practical success which the meeting at Grosvenor House on April 28th proved to demonstration.

There is reason to fear, however, that the work of the association may be impeded by the action of certain impatient people in small towns and districts, who, finding it difficult to obtain the services of a thoroughly trained nurse, are striving to hurry up the movement by employing someone whom they call a nurse, but who may have spent but three months in a hospital or some institution, a period so short as to barely enable her to find her way about the building with reasonable facility. These good people are mistaken in their views and aims, as it is quite impossible for anyone to do the work of a district nurse who has really no knowledge, to any practical extent, of the duties which devolve upon her in such a capacity.

Nor is this all, for it is a misnomer to apply the term "nurse" to anybody who has not obtained a thorough training in all the duties of the office, and these three-monthly cottage helps, if dignified with a name to which they can lay no claim, must create a prejudice in the minds of the poor, by reason of their inefficiency and absence of knowledge. It is to be hoped, therefore, that the members of the general public in every district of the country where the demand for a district nurse may arise, will decline to provide the money for the support of anyone who is not thoroughly trained, and in every way capable to discharge the duties of this responsible office.

The advantages of a trained nurse to a district are manifold and obvious. She speedily introduces neatness, order, and method into the cottages of the poor. If she is to be successful, she will be found to exhibit three main qualities, namely, intelligent obedience to the doctors' orders, a love for the work, and great commonsense. In large towns where hospital accommodation is ample, operations of a serious character are seldom or never performed in the houses of the poor. In country districts, however, so soon as a skilled nurse obtains the confidence of the doctors, she proves an enormous help to patients and practitioners alike; the doctors, with her assistance, find it practicable to undertake operations in the houses of the poor which they never could attempt without the presence of the district nurse.

Many nurses are inclined to think that chronic cases are very tedious and uninteresting, yet untold misery is entailed in a small household by a case of cancer where no nursing worthy of the name is obtainable. In such chronic cases as these the nurse, by her knowledge and skill, can not only render the life of the sufferer relatively happy, but she can maintain a purity and sweetness in the atmosphere, renderino such cases almost, if not quite, unobjectionable, where, without her aid and attention, the lives of the healthy members of the family might become a burden almost too grievous te be borne.

In a large number of medical cases, too, the district nurse is able to render inestimable service to doctors and patients by the skilful administration of medicine, the preparation of baths, and in a hundred other ways. In obstetric cases, also, the trained district nurse is invaluable. She diminishes suffering, preserves life, and makes the work of the general practitioner infinitely less vexatious and much more liveable than can readily be understood by those who have no experience of such cases in poor districts.

The privileges of a trained nurse, too, are considerable. In this country at the present time there must be at least 10,000 trained and certificated nurses of the highest efficiency. They constitute a great civil service of women workers who have won their way to public respect and deserved honour. They now possess by their own forethought and energy a great nurses' co-operation, which gives to private nurses practically the whole of their earnings, and these earnings, in the case of that one co-operation, amount to quite £20,000 per annum. They have, besides, the security of the Royal National Pension Fund for Nurses, which enables any nurse who has sufficient thrift and foresight to join that Fund, to place herself in a position of absolute security against the day of sickness or incapacity from any cause. If a nurse joins the Pension Fund and takes out a sick pay policy, she may make her mind perfectly easy so far as this world is concerned ; and the Fund has besides a benevolent branch, of which Lady Rothschild is the able and energetic President, which is strong enough to give to every member of the Fund the maximum of security against sickness, incapacity, and old age.

It is amusing, though not surprising, to notice that a medical paper has been so struck by the success of the Pension Fund as to state that it was a little hard that nurses should have thus received adequate protection through their own exertions and the co-operation of the merchant princes of the City of London, when the great medical profession had comparatively nothing like it to show in their own defence. This is scarcely correct, however, because Mr. Ernest Hart has established a medical sickness and annuity society, which is strong enough to meet all the needs of the members of the medical profession if they care to avail themselves of it.

There is another aspect of this question that deserves more attention than it has heretofore received. It is the duty which the inhabitants of a district owe to the trained nurse who renders such essential services to all classes of the community resident within it. The district nurse is entitled to receive proper pay and suitable lodgings, and ample means of locomotion. Every week, however, attention is called to the circumstance, that there are very many people who think it ought to be possible to obtain a trained nurse for the payment of from £30 to £50 per annum, with the addition of indifferent lodgings. This is a wholly wrong view to take, and no district ought to think of employing the service of a district nurse unless they are prepared to pay her at least £70 a year, and to provide her with a separate sitting-room and sleeping-room, and also with fuel and light. It is further necessary to impress upon the well-to-do inhabitants of every district which employs a district nurse that they owe her an amount of personal service which it would be as honourable in them to tender as in her to thankfully accept. The trained nurse, being an intelligent and capable woman, needs society, exercise, and recreation. Those who have carriages might very reasonably arrange to give the district nurse a drive, say once a week, and to see, by an arrangement amongst themselves, that she is provided with some society and ample recreation. This view of the personal service which the well-to-do owe to the nurse is one which requires to be driven home to the minds of all who are in a position to offer such reasonable hospitality and sympathy to the devoted woman worker, who lessens the burden of their individual responsibility by ministering to the pressing needs of the suffering poor. District nursing in the highest and best sense, as represented by the members of the Metropolitan and National Nursing Association, embodies a great reforming movement, fraught with momentous issues to the poor and lower middle classes of our day and generation. The district nurse, in the course of her duties, inculcates principles of thrift and self-help, teaches sound sanitation, and prompts self-respect to an extent which at present is little understood and appreciatsd by the majority of people.

In a few years' time, when the work and teaching of the district nurse have had time to make their influence felt widely among the poor, there can be little doubt that the great out-patient question, surrounded as it is by difficulty and abuse of various kinds, will gradually reform itself, because the individual citizen will take a more intelligent interest in conserving his own honour, and so hesitate to accept free medical relief if he can by any means afford to pay something for it.

The association is admirably officered; during last year at the first International Nursing Congress at Chicago, which was attended by hundreds of nurses, amongst whom were the most energetic and able of the leaders of nursing in the United States of America, Miss Hughes, the Superintendent of the Metropolitan and National Nursing Association, represented English nursing in a manner which redounded to its credit, and was universally recognised by all who were present at the Congress. English nurses had few representatives at Chicago, and it is no exaggeration to say that Miss Hughes, during the discussions which followed the reading of the papers, took up and maintained the position of an English lady, full of knowledge, with great tact and skill, and so proved herself a representative of whom England might indeed, and justly, be proud.

### Appointments.

[It is requested that successful candidates will send a copy of their applications and testimonials, with date of election, to The Editor, The Lodge, Porchester Square, W.]

Carnarvonshire and Anglesey Infirmary, Bangor.—Miss Annie M. Paynter has been appointed Matron of this infirmary. She entered the training school in connection with the Liverpool Royal Infirmary in 1885, and for the last five years has been a Sister at that institution. Miss Paynter has excellent testimonials, and takes our best wishes and those of all who know her to her new work.

Royal Alexandra Hospital for Sick Children, Brighton.—Miss E. Fraser, who was trained at Guy's Hospital, has been appointed Matron of this hospital. Miss Fraser had charge of wards at the Norfolk and Norwich Hospital, and was afterwards Sister of the Somerset Ward at the Seamen's Hospital, Greenwich, and we cordially congratulate her on her appointment.

### Royal National Pension Fund for Nurses v. Record Press (Limited).

We have received the following letter from Mr. T. H. Willett, of Howard House, Arundel Street, Strand, the solicitor to the defendants in this action. It will be seen that Mr. Willett describes himself as "the proprietor of the *Nursing Record*:"—

Howard House, Arundel Street, Strand,
London, W.C., 1st May, 1894.

Dear Sir,—I notice that in your last issue you print the article relating to the Royal National Pension Fund for Nurses which appeared on the 14th ult. in the *Daily Chronicle*, and as the same is misleading and incorrect, the action for libel was brought against the Record Press (Limited), in respect of statements which appeared in the "Nursing Directory" and "How to Become a Hospital Nurse," the periodical the *Nursing Record* was not involved in the matter at all, and as to this I would refer you to the statement of claim delivered in the action, which I believe you are cognisant of. I must request you, as the article is likely to cause me, the proprietor of the *Nursing Record*, serious injury, that you insert in your next issue in an equal conspicuous position, a copy of the correction as the same appeared in the *Daily Chronicle* yesterday, together with an apology, otherwise I regret I shall have to commence proceedings in respect to the same.—Yours faithfully, T. H. Willett.

The Editor, Hospital.

The correction in the *Daily Chronicle* of April 30th to which Mr. Willett refers was as follows:—

In a recent article on an action between the Royal National Pension Fund for Nurses and the Record Press (Limited), we referred to the *Nursing Record* as the defendants. The mistake was due to a hasty reading of the statement of claim, in which an extract from the advertisement columns of the *Nursing Record* was incidentally referred to. That journal, however, had no association whatever with the complaint of the directors of the Pension Fund, and we are sorry that the confusion arose.

### PENSION FUND FOR NURSES.

The following letter from the plaintiffs' solicitors appeared in the *Daily Chronicle* of May 4th:—

Sir,—You will remember that the libel sued upon in this action, on which an excellent leading article recently appeared in your columns, was a published statement that the Royal National Pension Fund for Nurses "charges nurses from twenty to twenty-six per cent. higher premiums than they would have to pay for similar advantages at old-established London offices." The paragraph published in your issue of yesterday states that the *Nursing Record* "had no association whatever with the complaint of the directors of the Pension Fund." May we, as solicitors to the plaintiffs in the action, call your attention to the following passage in a leading article, under the heading of "The Royal National Pension Fund for Nurses," published in the *Nursing Record* of Jan. 28, 1892 (page 75)?—"We furthermore ask whether it is justifiable that a Royal National Pension Fund—munificently endowed—should charge a poorly-paid class of working women from twenty to twenty-six per cent. more than they would have to pay if they applied for the same benefits at an ordinary insurance office."

Again, in the *Nursing Record* of August 10th, 1893, an advertisement appeared as follows:—

"Annuity Fund and Sick Pay Fund for Nurses.—Figures for nurses to note and remember.—For the same annuity nurses have to pay from twenty to twenty-six per cent. more to the Royal National Pension Fund for Nurses than old-established and very wealthy insurance offices demand. . . . Every nurse interested should send a stamped addressed envelope to the Editor, the *Nursing Record*, 376, Strand, London, W.C., for full particulars."

In the face of these publications it can hardly be said that the *Nursing Record* had no association whatever with the complaint of the directors (council) of the Pension Fund.—We are Sir, your obedient servants,

Baker and Nairne.

3, Crosby Square, London, May 3rd.

In reply to Mr. Willett's letter to the Editor, the solicitors for The Hospital wrote to him saying that the correction and the letter of the plaintiffs' solicitors would be republished in this issue of The Hospital, and that they were instructed to accept service of legal process.

### The Wardroper Memorial.

The Archbishop of Canterbury has kindly revised his address on the occasion of the dedication of this memorial. See The Hospital for 5th inst., page xliv.

Address by the Archbishop of Canterbury.

It is probably impossible for the majority of the present generation to estimate at its true value the work done by two noble women, Miss Nightingale and Mrs. Wardroper. The nursing and general efficiency of the hospitals at the present time are such that very few now living can appreciate the difficulties which beset Mrs. Wardroper forty years ago, when nurses were untrained, and were mostly drawn from the lower strata of society. At that time, as a young clergyman, he was in Rome, and had some conversations with a great and intelligent layman of those days about the Crimea and the war. He dwelt upon the new devotion displayed by the band of thirty-seven nurses who went to the seat of war under Florence Nightingale. The layman expressed surprise that he should speak so strongly on the point, and added that without an organisation of this kind he could scarcely think that the work of the Church of England itself was at its true height. How changed was all work of this kind since that date. He, the Archbishop, felt it a privilege to bear testimony to the enormous influence for good which the work of Florence Nightingale had had on the Church of England as a striking fact. He remembered the sensation created on receipt of the news that this devoted band of nurses had landed in the Crimea on the very day of the battle of Inkermann. History would do justice to Miss Nightingale's vigour, great judgment, and mastery of her subject. The masculine power with which she expounded her plans to statesmen and generals was remarkable, whilst her gift of administration soon produced discipline, comfort, and sanitation amongst the sick. Nor was this all, for the moral influence of her work speedily made an impression upon the people at home. Its effects were remarkable, they produced a revolution in the care of the sick, secured the intelligent co-operation of hosts of good women, and were felt throughout the whole of the British Empire. It was fitting that St. Thomas's Hospital dedicated to Thomas-à-Becket, with its venerable history, should have worthily become the home of the organization for training which then took its rise. He felt that St. Thomas's Hospital came home when it came to Lambeth and occupied a site within view of the White Tower of Lambeth Palace. It was a pleasure to come over to this great building, which he saw every day, and to speak to the memory of her who was preparing for Florence Nightingale's work at home during those fateful months in the Crimea. How great was the blessing upon Mrs. Wardroper's work, who in quiet resourcefulness was reforming and conforming nursing, and preparing the way for better things, until when the nation awoke to the character of Florence Nightingale's work, and subscribed £50,000, St. Thomas's Hospital was ready at once to make that memorial a practical success.

What her work was was most fitly touched in that prayer of John Keble's which had just been used in the service. What a blessed career, indeed, was that of Mrs. Wardroper, who, with all her heart, devoted the best years of her life to great works of mercy and loving tenderness to the sick poor! How great the moral and spiritual influences of a religious spirit in hours of suffering! How blessed is the work of such a woman, how far-reaching its results, how great its usefulness, with 500 nurses and 50 matrons trained by her own master-hand, and distributed throughout the world to act as centres of training and distribution for thousands of other attendants upon the sick! How simple and loving was the nature which led her, after the toil of the day, to spend hours in decorating this very chapel, which she loved so well, for festivals and high days, a chapel which by this memorial of her will be decorated for ever! Her nurses and their successors will pass and repass it constantly, attended by the blessings of dying men and the gratitude of the living. Edward VI., the second founder of the hospital, on his death-bed, said: "Lord, I thank Thee that Thou has given me life thus long to finish this work to the Glory of Thy Holy Name!" So will we part with her we commemorate to-day, who might have repeated as her last words those uttered by Edward VI., and say to her with Florence Nightingale: "And so, dear matron, as thou was called so many years, we bid thee farewell, and Godspeed to His higher world; not as the world giveth, giveth He thee."

### Queen Victoria's Jubilee Institute for Nurses.

The report of the council of The Institute has been laid before the Queen, and it appears in every way a satisfactory one, 322 nurses being at present on the roll. Many new districts have been affiliated during the year, making a total of 119 associations in England (including rural), Scotland 49, Ireland 12, and Wales 12.

The work of the inspector is increasingly valued by the affiliated associations, and it has evidently aided, in many cases, in raising the standard of nursing. The systematic supervision of a trained nurse, unconnected with local committees, must be distinctly beneficial to the workers.

The Scottish branch, under the presidency of Her Royal Highness Princess Louise, continues remarkable for energetic good work; and the Irish and Welsh branches are making excellent progress. The Rural District branch, which is under the general superintendence of Miss Oldham, also does much valuable work, dealing with all places with a population under 9,000. The report calls attention to the ever-increasing demand for nurses, a demand which is very difficult to meet, although The Institute continues to do most important work in training and supplying as many as the funds permit, yet, whilst new associations are constantly applying for advice and assistance, it is most difficult to comply adequately with all demands.

### Minor Appointments.

St. Mary's Day Nursery and Hospital, Plaistow, E.—Miss Newport has been appointed Matron of this institution. She was trained at the London Hospital, and was afterwards Sister at the Brompton Hospital for Consumpton; Head Night Nurse at the Chelsea Hospital for Women; Head Nurse at the Kendal Hospital; and Sister for over three years at the West London Hospital. We congratulate Miss Newport on her appointment.

### Where to Go.

St. Thomas's Hospital, Westminster.—On June 9th, at four p.m., the recent additions to the Medical School will be formally opened by H.R.H. the Duke of Connaught.

### Wants and Workers.

Any information regarding a holiday home for nurses in Brussels would be gratefully received by *Nurse E.D.*

Will any lady or gentleman kindly lend a full-sized bath-chair to a young man suffering from advanced phthisis for immediate use? Or is anyone willing to part with a second-hand chair (wicker with india-rubber tires preferred) on very reasonable terms for the above case?—*Miss Helen Clements, The Sub-Deanery, Lincoln.*

### Our Annual Leave.

### A HOLIDAY IN SARK.

On the Way to Sark.

Sark? Most people have to hunt back through their memories until with the recollection of their school days comes the once familiar sequence of names, "Jersey, Guernsey, Alderney, and Sark." "Of course, the smallest of the Channel Islands." But no such thing, for when you go to Sark you

Entrance into Sark.

will find many other islets in the group, notably Herm, Jethou, and Breckhou, all well worth a day's excursion. We enjoyed our stay in Sark so much that we are anxious to let other nurses know what a delightful holiday can be spent there at moderate expense. It is not a fashionable place by any means. There is one shop, no railways, cabs, omnibuses, cycles; no postmen, papers, or telegrams; no noise, no hurry; and for the over-worked nurse and tired brain-worker who longs for a pause from daily work or worry, no place is better than Sark. The best way to get there is from Waterloo to Southampton by the boat train starting in the evening. If you mean to go straight through to Sark, you must arrange your journey so as to arrive in Guernsey the day the small steamer makes the trip, for it only goes three times a week.

The Channel steamers are very good, and leaving Southampton Docks about midnight arrive at St. Peter's Port, Guernsey, between six and seven the next morning. The journey is broken here, and as the Sark steamer does not leave for some time after the London boat gets in, it is well to have breakfast at one of the hotels overlooking the harbour. Then if you have good fortune, as we did, you will see the sun rise over Sark in the distance; a wonderful spectacle, which it has been a joy ever since to look back on.

The little steamer, which seems such a cockle-shell after the other, makes the distance in a little over an hour; the short passage is exciting owing to the many rocks and shoals which render the course very devious. Sometimes the steamer appears bound for the open sea, then it swings round apparently bent on wrecking itself on one of the rocky islets in the way; baulked in this endeavour, it makes a rush towards a narrow passage between two huge rocks. Every passenger who has not previously made the journey is aghast, and ready to believe that the opening is several feet too narrow for the vessel to squeeze through; however, it is done, for there is room and to spare! There is one curious Martello tower which looks as if it were built in the sea.

When the steamer at length drops anchor at Sark, the passengers are confronted by an inaccessible granite cliff, up which is no semblance of road or footpath. Disembarking from the steamer into small boats, they are rowed round the arm of a small breakwater (jutting out from the cliff like a capital J) into a miniature harbour, and then the secret of entrance is disclosed, for the interior of the island is approached by way of a small tunnel, *through* the cliffs, not over. The island is small and apartments scarce, so that it is best to write for rooms beforehand; several of the small farmers take lodgers, and there are also two very good hotels, Dix Cartis and Bel Air.

Apartments are not dear. We paid 25s. a-week for two bed-rooms and a sitting-room, but provisions are expensive. For instance, butter 1s. 10d. a pound; a very small jar of

The Coupée, Sark.

gooseberry jam, 1s.; butcher's meat was dear, too, but poultry and rabbits quite reasonable. Perhaps it would be wise to supplement the resources of the island by taking potted meats, preserves, &c., over from Guernsey.

Our experience of "the shop" was that it did not contain much variety, its chief commodities appearing to be bread, sardines, sweets, and fish hooks, but then it was out of the season, so perhaps its capabilities are expansive.

There is a post office in Sark, but both the inward and outward mails are greatly dependent on the weather, and if the sea is at all rough, landing is impossible. We were told that often in the winter there could be no communication with the outer world for a week or fortnight at a time.

The air of Sark is pure and invigorating, and there is a variety of climate, from the breezy downs aglow with amethyst ling and purple heather, where, no matter what quarter the wind is in, it blows always fresh from the sea, to the cosy nooks among the rocks, in which one can bask all day in sunshine, watching the black cormorants fishing and the curious rock fish swimming lazily through the floating trails of crimson and amber seaweed. The water is so crystal clear that it reveals much of the beauty and mystery of submarine life and its luxuriant vegetation.

In exploring the coast one comes unexpectedly by winding passages and through odd little tunnels into the loveliest coves imaginable, where it would not seem so very astonishing to find a beauteous sea maiden reclining on a rock and combing her locks with a golden comb.

The Gouliot caves are most interesting, full of rare zoophytes and lovely seaweeds; they can only be approached at low tide, and it is quite necessary to have a local guide.

The celebrated Coupée is a natural bridge connecting Great with Little Sark. When first we crossed it we walked in single file, keeping our eyes carefully on the centre of the road, fearing to look an inch to the right or left lest a sudden giddiness should make us stumble over the frightful precipice which yawns on either side, but familiarity bred contempt, and our last expedition in Sark was made to see the Coupée by moonlight.

One hint more: wear old things; the inhabitants are not critical, and in jumping stone walls or clambering up and down the steep cliff paths it is as well to know it won't matter if your dress is spoilt. Above all don't put on good boots; they should be water-tight because of the heavy morning dews, but boots worn in Sark can never be used afterwards, for the granite cuts them to pieces.

### Technical Education Committees and Nursing.

The Hon. Secretary of the Dorset Health Association writes as follows: There has been lately so much misconception as to the limitations under which the Technical Education Committees may promote the subject of "Nursing," that it may be useful to the general public to have a little more accurate information on the question, than that which has hitherto prevailed. Having done me the honour to mention my name some time back as "original promoter of the proposed technical nursing scholarships," up to quite recently under debate it may interest your readers to hear the result. Convinced that in some counties, the ardent advocates of the nursing cause, were far outstepping the limitations of the Technical Education Acts by their proposals, I sought and obtained an interview on the subject at the Science and Art Department, South Kensington, in order to ascertain if I had unwittingly done the same, in the matter of the proposed scholarships. Aware that, paradoxical though it may appear, as we stand and survey some of our technical classes at work, that technical money may not be used for "teaching a trade or industry" (see Technical Instruction Act of 1889, clause 8), I was equally alive to the fact that the idea of using technical money to give the regulation three years' hospital course (laid down by the British Nurses' Association, as the test of the profession), was manifestly contravening this limitation. On the other hand, I urged that "courses of special instruction in the principles of nursing," in which local nurses were often deficient, and which were not to exceed the limits of a three or six months' course, under the head of "Scholarships," would fall well within the provisions of the clause relating to "scholarships" in the Act of 1890. This would surely be as permissible as to give similar "courses of instruction," as is now done, to local dairymaids, musicians, engineers, or cooks in certain technical details of their trade in order to perfect their execution. The result of my interview was a suggestion, that I should embody my views in a formal memorandum, which I did, and to which an answer was duly returned me, after the matter had been given a careful consideration at headquarters. I was told that I should be justified in renewing my application to the County Council under one or other of the following heads: 1. "In aid of courses of lectures on nursing or in respect of classes for teaching nursing," such as were described in my memorandum A. 2. "For the establishment of scholarships" (B), under the provisos I had stated, viz., so long as they were "open to everyone in the county" (*i.e.*, "open to local women," as I had worded it), and "were held for instruction in the principles of nursing, and not for such practical work as would enable the holders to become adepts in any particular branch of the profession," and "provided the institutions at which the scholarships were tenable were not conducted for private profit," which I had also stated. To this I replied by saying, that we should not pretend in a course of three or six months, to make our local nurses "adepts," or to enable them in any way to compete with a professional hospital-trained nurse, though the said course of instruction would immensely increase their usefulness. In reply to a question as to whether a three months' maternity course—enabling a woman to pass the L.O.S.—would be permissive, or would render her an "adept," the department was of opinion that this would be clearly outside the provisions of the Act, and to this I am reluctantly obliged to agree. It will therefore be seen that nursing may not be taught by means of technical money, as a complete profession from beginning to end, but that defects in such an education may apparently be remedied by judicious "courses of instruction," and thus ignorant practice may be remedied by intelligent principles. That district nurses may not be maintained out of technical money (as some enthusiastic ladies have averred) is perfectly self-evident, though their services as teachers of the principles of nursing may no doubt be remunerated, and a grant in aid of such teaching may probably be made. In reply as to whether a grant in aid of a Home or Institute at which such scholarships were tenable, was permissible, the reply was to the effect, that if not conducted for private profit and if eligible to receive scholars, it would probably be eligible to receive some such grant for the technical instruction carried on there. To recapitulate, it will thus be seen that money may be voted—(*a*) To aid lectures or classes such as those held by the National Health Society and the Sanitary Institute. (*b*) To give nursing scholarships under certain conditions for courses of instruction in the principles of nursing. (*c*) That grants in aid of a county Home, where such scholarships are tenable, may be also applied for, under certain restrictions applicable to similarly assisted schools or institutions. It will thus result that it will be local women who in each county will chiefly reap the benefit of the instruction, women whose ignorance is rather their misfortune than their fault, and who can never for a moment, even after the said course of instruction, be confounded with the heads of the nursing profession, who have gone through the whole curriculum from beginning to end and are entitled to class themselves as "the professional nurse." In conclusion, I begged that it might be understood that a "local" or "cottage" nurse is considered at least in Dorset, to be one who has received "a course of instruction in the principles of nursing," but not such as to fit her to be considered an "adept" in any branch of the profession.

### The Muses' Looking Glass.

### EXHIBITION OF THE ROYAL ACADEMY.

Certain comments on the part which finances played at the New Gallery hold good as regards the present exhibition at Burlington House. Indeed, with certain marked exceptions, this comprises, for the most part, portraits, which represent "orders," and pictures painted with a keen sense of the marketable. Throughout the gallery there is a decided absence of venture and daring, a certain clinging to the requirements of the public; a following rather than a leading of public taste.

The President contributes six examples of his work, in which he shows himself uniformly at his best, in technique certainly, if in a lesser degree in subject. "A Summer Slumber" (No. 111) is a charming study of a beautiful girlish form reclining. The whole picture is suffused with a sense of silence and repose, which has a tranquilizing effect on the spectator.

The solitary little figure lies sleeping on a marble slab, a wealth of dishevelled hair around her. The warm drowsy light—the atmosphere within the marble building, and out beyond, in the decorative landscape, is a masterpiece of successful treatment, delightful in execution as in suggestion. "Fatidica" (No. 20), of smaller dimensions, does not present quite so striking an instance of Sir Frederick Leighton's skill. It treats a sibyl reclining in an antique cathedra. In "The Bracelet" (No. 135) he shows another female form standing; and in "The Spirit of the Summit" (No. 190), in the Great Room, again another sitting. High on a mountain crag she sits, pinnacles rising around her, and above her head a deep vault of blue sky.

Mr. Luke Fildes' portrait of the Princess of Wales hangs on the south wall of the same room as the "Spirit of the Summit." It has evoked a certain amount of criticism on account of its background of drapery, but it also attracted much attention on its own merits at the Society's private view on Friday. The Princess is seated on a tapestry sofa, a toy terrier under her right arm. Her dress is of some black material, through the semi-transparent sleeves of which her arms are discernible, finely modelled. Mr. Luke Fildes has two other studies, but this is the principal work which he exhibits this year.

In a conspicuous manner Professor Hubert Herkommer distinguishes himself in the large canvas (No. 340) which he calls "All beautiful in naked Purity." In this work, of elephantine porportion, there is nothing of the artist as we have hitherto known him—nothing but the signature in the corner. It is true his versative mind has bewildered us more than once, but never has he shown himself "more unlike himself" than in the present instance. The woman's figure, her entourage, the elaborate detail, the trailing biar roses, they are fresh to us, coming from this particular source. Therefore the canvas is an astonishment to us rather than a picture, and we refer to the other examples of Professor Herkommer's brush, as exhibited in certain portraits which monopolise, if they do not adorn, the walls of the gallery. Nearly all of these, if not quite all, are treated to backgrounds of a markedly warm inclination, red or crimson, or madder brown. The same background does not suit all subjects, but this the Professor appears to forget. The question of background is of so all-important a value that no artist can afford to overlook it. Mr. Shannon shows us in a positive manner this which Professor Herkommer shows us negatively.

Mr. Solomon's portrait of Mrs. Patrick Campbell has been by no means overrated. It is a masterpiece, as is also a smaller portrait which he contributes of Mr. Zangwill. Both Mr. Solomon and Mr. Orchardson, much to our regret, have painted no dramatic composition this season, but both alike in their portraits show us in their respective, if opposite mode of work, that neither mind nor brain have been inactive. Very clever and very striking is a three-quarter study of female portraiture in the case of the latter. This particular "Portrait of a Lady" Mr. Orchardson gives us in monochrome. It is both an original and a daring venture, and the artist has been successful where others less skilled would have failed, and the painter's complete mastery of "Valeur" is depicted here in a telling manner. Somewhat prolific is Mr. Sant in his canvases this year. He is uneven in his work, and whilst in one instance—and this a portrait—he surpasses what we have been led to expect of him by his previous pictures, in "The Pastural" this is by no means the case. Here he shows himself at his worst. Two small, self-conscious, unchildlike children are sharply defined and cut out against surroundings with which their figures are in no apparent harmony.

Gallery VIII. contains what is perhaps regarded as *the* picture in the collection. It is a very large canvas by Henrietta Rae, in a central position on the west wall. The subject thus represented is "Psyche before the Throne of Cupid," but in reality it is little but a study of beautiful females forms, beautifully grouped, and as such, may fairly be ranked among the monumental works of the age. In general scheme and harmonious grace it will find itself without rivals. It may not lay claim to being a great or a suggestive work, which appeals to one's intellect, but it does lay claim to pleasing the eye by its freshness and the happy manner in which it is painted.

Other subject pieces from Mr. Poynter display all the realistic accuracy as to detail which have been the result of a lifetime's application. Several very highly finished, conscientious works of this artist are to be found in the various rooms. "À Versailles" by Mr. Val C. Prinsep is another instance of supreme subordination to minutiæ. Here, the individual treatment of his figures detract in a considerable manner from the breadth and dignity of the composer's subject. The figures, besides, are too large and too crowded for the proportions of the canvas, neither do they suggest the abandon—the spirit—and life of a great national movement, such as they are meant to represent.

Mr. Frank Bramley in "The Light of the Fire" (No. 539), has given us a consummately good study of fire-light effect. This is one of the striking pictures of the collection. The sad and morbid tone of the artist's previous work is here notably absent. A happy-faced babe with round cheeks, half in shadow and half in brilliant light, seated on its mother's knee, is the centre figure of the group. The light from the fire is never exaggerated nor overpainted, it just catches, as we have often seen it catch, accentuating certain points in the surroundings.

"At the Eleventh Hour" is dramatic if nothing else. But it is something else. It is what one might fairly call a speaking picture; the contrast of the bride with the happiness of her surroundings, is at once artistic and poetical. The girl's attitude in all its hopeless defection—the mocking sunshine without, as within, the laughing faces contrast forcibly, and the picture is one which appeals to one in a very marked way.

Landscape is not so much to the fore this year, at least not good landscape. Seapieces are best represented by Henry Moore and Napier Henry. Landscape by Leader, Macbeth, and Wyllie. The statuary shows nothing of monumental value. A melancholy interest attaches itself to the sketch-model of the tomb of H.R.H. the late Duke of Clarence. This is executed by Mr. Gilbert, and the design, taken as a whole, is a triumph of any age

### Maternity Work in U.S.A.

NURSERY AND CHILD'S HOSPITAL.

Such is the inscription over the door of one of the best lying-in hospitals in New York. This charitable institution was founded by a wealthy lady and is supported by voluntary contributions, and is now under excellent management.

To describe the building we will pass through the polished hall and up the wide staircase and begin at the top floor, which is divided into a long ward containing some twenty beds, two smaller wards each containing six beds, a scullery, cupboards, and bath-rooms. Between the two smaller wards there a little room called the delivery-room.

Here all is in perfect order, and nothing in service in this room is ever used elsewhere, basins, receivers, bed baths, douche cans, &c., all are of enamelled iron and quite sound—at the first crack they are banished.

Downstairs is a room where the women wait until with the first "pain" they are transferred upstairs and the doctor is sent for. They are in the care of experienced nurses.

The bed in the delivery room is rather high and hard. First it is covered all over with a mackintosh sheet, then the sheet and drawer sheet, and over this a thick pad. When the time is close at hand an electric bell summons the doctor.

The baby when born is received into very thin paper, as this makes it easy to lift, and is then weighed. The head nurse afterwards takes special charge of the mother, whilst the second nurse removes all stained linen and washes it. The baby in the meantime is rolled in a blanket.

After six hours a truck bed is brought to the side of the delivery bed and the woman is rolled over on to it and is then wheeled into one of the smaller wards and put on a clean bed, has a warm drink, and then the baby is washed and given to her.

After each confinement the delivery-room is scrubbed with carbolic and every utensil is disinfected and scalded in boiling water. The smaller wards are disinfected with sulphur every third week. At the end of a fortnight, if all goes well, the patient is transferred to the large ward. Married women or girls with their first baby only are admitted. They can either pay £5 down, which enables them to remain in the hospital until the doctor considers them quite well, or if they cannot afford to pay they have to work for the hospital for three months after they are pronounced well. The work con-consists of cleaning or cooking, or they may have to mind and nurse another baby with their own.

Situations are got for them when possible as wet nurses, and under certain conditions other situations, and then they pay so much a week for the hospital to keep the baby for them. The baby and mother after a month, if strong enough, are sent down to the next floor, when the mothers do all the work, but there is a nurse to superintend them. There is a school attached to this institution where the children are sent at three years old, their mother or some friend guaranteeing payment. There are also private wards where patients of a better class are received.

I am afraid that this slight sketch does not do nearly justice to the good management, order and thoroughness of this most excellent institution, but, such as it is, I hope it may interest some readers of The Hospital. A. E. I.

### Notes and Queries.

**Queries.**

(55) *Sisterhoods.*—Can I join an Anglican sisterhood and continue to go out to private cases?—*Lady Nurse.*

(56) *Male Nurses.*—Where can a young man be trained as a nurse?—*A. E. D.*

(57) *Training.*—Is there any chance of a girl of twenty getting admitted to a hospital training school?—*E. P.*

(58) *Medical Women.*—Where can I obtain information about medical training for women?—*A. E.*

**Answers.**

(55) *Sisterhoods* (*Lady Nurse*).—You had better address your question to the heads of the various sisterhoods, whose addresses you will find in "Burdett's Hospital Annual."

(56) *Male Nurses* (*A. E. D.*)—We regret that we know of no institution in England which gives a full training in general nursing to men.

(57) *Training* (*E. P.*)—You are too young. Read "How to Become a Nurse," by Honnor Morten.

(58) *Medical Women* (*A. E.*).—See The Hospital of September 10th and September 17th, 1892, for articles headed "How to Become a Medical Woman."

### For Reading to the Sick.

REST IN WEARINESS.

**Motto.**

Absence of occupation is not rest.—*Cowper.*

**Verses.**

Art thou patiently toiling, waiting the Master's will,
For a rest that never seems nearer, a hush that is far off still?
Does it seem that the noisy city never will let thee hear
The sound of His gentle footsteps, drawing, it may be, near?
Does it seem that the blinding dazzle of noonday glare and heat
Is a fiery veil between thy heart and visions high and sweet?
What though a lull in life may never be made for thee?
Soon shall a better thing be thine—the Lull of Eternity.

—*F. R. Havergal.*

O wakeful toiler in a world of pain,
A long rest waiteth thee;
Seek it not here, but bravely lift again
Tired hand and feeble knee.
If thou wilt trust, thy Master will sustain;
And as thy days, thy strength shall be.
The Father portioneth, as He will,
To all his beloved children—and shall we not be still?
Is not His will the wisest? Is not His choice the best?
And in perfect acquiescence is there not perfect rest?

—*F. R. Havergal.*

**Reading.**

Surely my heart cannot truly rest, nor be entirely contented, unless it rest in Thee, and rise above all gifts and all creatures whatsoever.—*Thomas à Kempis.*

Thou has made us for Thyself, and our hearts are disquieted until they can find rest in Thee.—*St. Augustine.*

The great disappointment to the convalescent is the short-lived hope of speedy recovery. If at first a great stride has been made, and health is seemingly coming very quickly, the feeling of joy which the first glow of returning health brings is very soon quenched.

In exchange for the feeling of exhilaration comes exhaustion, and consequent depression.

Instead of finding the marked improvement of the first days continue, it seems as though you can do positively less each day, or, if not less, yet you remain stationary. All the temptations which seemed buried during your illness (but which were, in fact, only slumbering) now return with double force. . . . Your full strength is very far off, and each little fatigue is a very real one. Dissatisfied with yourself, your progress towards recovery, your progress in religion, you give way to irritability and fretfulness. Much of this is due entirely to physical causes. With a weakened form and shattered nerves you have to make the effort to return to the daily duties of life, and feeling unfit for work of any kind, you must return to the busy world. To rouse yourself seems next to impossible . . . and the knowledge that in spite of all you must struggle on, is well nigh unbearable. . . . "Suffer not," writes Abbé Perreyve, "the long and uncertain continuance of your weakness to sadden or offend you. Do not give way; be not discouraged; endure, and await God's help. For why should you desire to know the issue of your sufferings? If you were assured they would end in death, in your present state of weakness you would lose all peace and quietness of mind. If, however, you were assured of speedy recovery, where would be the merit of your patient trust in God? Believe that all He does is for the salvation of your soul, and remain faithful in the midst of the deepest uncertainty."—*M. E. Granger.*

O merciful Lord preserve me. I am Thine, O save me; I am Thine, sanctify and preserve me for ever; that neither life nor death, health nor sickness, prosperity nor adversity, weakness within nor cross accidents without, may ever separate me from the love of God, which is in Christ Jesus my Lord.—*Jeremy Taylor.*

### The Book World for Women and Nurses.

[We invite Correspondence, Criticism, Enquiries, and Notes on Books likely to interest Women and Nurses. Address, Editor, The Hospital (Nurses' Book World), 428, Strand, W.C.]

The Voice of a Flower. By E. Gerard. (London: A. D. Innes and Co.)

In "The Voice of a Flower" we find ourselves walking in a world of unreality and romance. This is by no means without its pleasant side. Stories about barons and monster German castles appeal to the imaginative in our matter-of-fact existence. Count Wolfram Sturmfeder and a German castle are both here. The story opens disclosing him to our view, "standing on his own ancestral territory, surrounded by his vassals and tenants." He, of course, is the hero. Livia Ronsecco, who plays a leading part in the romance, is described throughout in superlatives—very young and very beautiful. Measured by her side other heroines are insignificant. In the course of the narrative this maiden's lover is slain by the hand of the mighty Wolfram, who lays his heart and wide domains at Liva's feet. A momentary failing in allegiance to the lost love finds the girl giving her promise to the Count; but it is not for long that he is destined to enjoy the fruits of his dearly-bought victory. When the tragic *dénouement* comes, his betrothed denounces him a murderer, Count Sturmfeder takes his life, and the beautiful cause of so much bloodshed retires into the secluded retreat of a neighbouring nunnery. In thus placing his scenes in foreign lands the author has fallen into an error others before him have fallen into, that is, laying too much stress on the various national characteristics conveyed through the portraiture of characters in fiction. In real life people have been known sometimes to depart from the traditions of their race—in novels this is never so.

The Rubicon. By E. F. Benson: (London: Methuen and Co., 1894. Two vols.)

Business is business and demand must be met with supply, therefore Mr. Benson has put into the market another "Dodo." Considering the success of his last novel, it would be as reasonable to abuse Mr. Benson for giving us "The Rubicon," as to remonstrate with Mr. Stead for writing "If Christ Came to Chicago." Both are journalism, and if we feel that Mr. Benson rather hides his undeniable literary light under the bushel of cheap popularity, after all it is his affair, and we are not his conscience. "The Rubicon" follows the social and mental career of one who is in most respects another "Dodo" in that by the end of the first few chapters Eva is married to the necessary but harmless member of the aristocracy. She flirts, says smart things, scores off her harmless husband, and ultimately falls in love with another man. Here the likeness ends, for the light novel readers of the declining years of the nineteenth century are not twice to be robbed of their prey. The British public has made up its mind and will stand no nonsense. Its satiated appetite demands that in the last chapter the Woman with a capital W shall die by her own hand. Therefore, on the last page of "The Rubicon" Mr. Benson, with unfailing good nature triumphantly supplies the prussic acid, and might well ask with conscious pride if his readers wanted anything more.

A Daughter of Music. G. Colmore. (London: W. Heinemann. 1894.)

In a "Daughter of Music" Mr. Colmore sustains the high literary reputation which his other novels have won him. He has again succeeded where many have failed in the endeavour to place uncommon people in uncommon situations. In his latest novel this is especially the case, and his characters interpret themselves in a manner which is entirely natural and artistic at the same time. Mr. Colmore has studied human nature to some purpose, and with him we study it too with somewhat awakened eyes. In his latest novel the writer treats the inconsistent side of life in a strikingly forcible manner. Thus he shows us his heroine with a two-sided nature, the one impelled by Anthony Dexter, the other subservient to the stronger impelling of Paul Garnett, whom she eventually marries. Her love for the former, engendered on her emotional and passionate side, conquers her better reason, and Rhoda leaves her husband's home and flies to southern scenes. The power of music over the human soul is here portrayed with no incompetent pen. It was to this passion in her complex nature to which her lover appeared and which conquered and ruined her. But it was apart from the stronger, steadier, more enduring love she gave her husband. Rhoda loved her husband all through, with the love a weaker nature yields to a stronger. She gave one side of her being, it is true, to Anthony Dexter, but the other, the finer, the more enduring, she gave, as she had always given, to the man whose name she bore. And Paul loved her, too, in an equally real manner, but neither saw into the heart of the other for many years after the fugitive's return. He forgave her in the sense that he received her back to the home she had deserted, but the forgiveness was little but a sham screen for the honour of his own name. The working out of his revenge, of which he had said it should not fail to sweep on through all Rhoda's life, took shape with a refinement of sustained cruelty, a relentlessness of purpose, which in its conclusion is by no means the least artistic part of the book.

### MAGAZINES.

The Contemporary Review for May contains, amidst much substantially good matter, a study of Mr. Gladstone by Richard H. Hutton. This is above all a study, not a rhapsody nor a vituperation, but as unbiassed a consideration of the merits and demerits of the great statesman as we have come across for some time. Mr. Hutton's recipe for the gratitude we owe the subject of his paper he sums up in the following manner. "Nor can we," he says, "I think, show our gratitude to him better than by helping to defeat the almost Titanic labours of his last eight years, and so to prevent it being said of him that after raising the whole level of English political disinterestedness, he set off against his country's immense obligations to him, the commission of the greatest and most disastrous blunder which ever neutralised the achievements of a splendid eloquence, an inexhaustible energy, and a generous enthusiasm as abundant as it was sometimes misplaced."

The Pall Mall Magazine for May is an especially interesting number. Paul Verlaine has the first few pages, in a quaint poem on Oxford. The poet's views find a spontaneous expression here on that grand old city, which impressed M. Verlaine in a genuinely serious manner. It was at the very time when he wrote this poem that he gave out he was "going to take London en route for Oxford." The hold the city had on him Paul Verlaine has explained in his verses. The poem is illustrated, but not quite adequately. "Unknown Paris" deals with student life in the French capital, and shows us much of the world of hardship and gay pleasure which centre round the artists' existence in their own particular Quatier Latin. "The Decline and Fall of Napoleon" is further continued, in unabated interest, by Lord Wolseley, and a short story of striking interest we find in "Julie" by Lucy Clifford.

An original and interesting article on "Hachisch Eating" appears in the Cornhill for this month. Haschish eating, this is by one who has a practical experience of it—and no pleasant experience it seems to be. To read this paper would act as a warning to anyone likely to indulge chronically in the fatal habit. The effect the drug has upon the brain is graphically described, and all the extravagant nightmares it brings in its wake. It reads very much as one would fancy a lunatic's condition of mind to be. "With Edged Tools" is continued, but not concluded; as is also "Matthew Austin," two serials which lay rival claims to our interest.

## Figures and Tables

**Figure f1:**
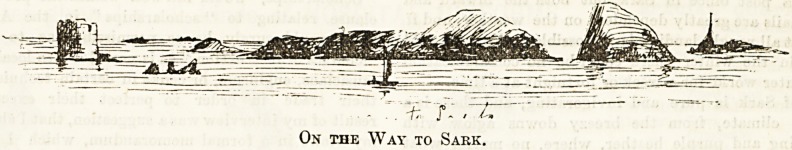


**Figure f2:**
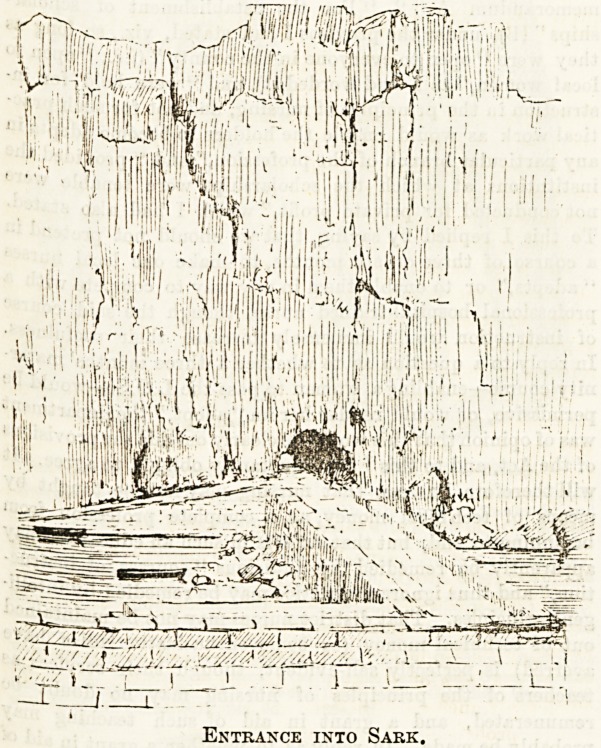


**Figure f3:**